# Participatory development of a home-based depression care model with lived experience older Nigerians and their caregivers: a theory of change

**DOI:** 10.1002/gps.6019

**Published:** 2023-11-01

**Authors:** Akin Ojagbemi, Stephanie Daley, Yvonne Feeney, Olufisayo Elugbadebo, Lola Kola, Oye Gureje

**Affiliations:** 1 World Health Organization collaborating centre for research and training in mental health, neuroscience, and substance abuse, Department of psychiatry, College of Medicine, University of Ibadan, Nigeria; 2 Centre for Dementia Studies, Brighton and Sussex Medical School, Brighton, UK; 3 Centre for Global Mental Health, Health Service and Population Research Department, Institute of Psychiatry, Psychology & Neuroscience (IoPPN), King’s College London

**Keywords:** Action research, Caregiver burden, Comorbidities, Community based participatory research, Ethnogerontology, Health disparities, Home health care, Low- and middle-income countries, mhGAP-IG, Psychosocial interventions, Task sharing, Stigma, Underserved populations

## Abstract

**Background:**

There is a huge treatment gap for late-life depression in sub-Saharan Africa. Building on prior work to scale-up mental healthcare with the aid of the WHO Mental Health Gap Action Programme Intervention Guide electronic version (emhGAP-IG), this study aims to involve older people in the iterative development of innovations to overcome challenges in the detection and clinical management of late-life depression by frontline non-specialist primary healthcare workers (PHCW) in Nigeria.

**Methodology:**

There were 43 participants in the study. We conducted formative qualitative research using 15 in-depth key informant interviews with persons who were 60 years or older and had a recent experience of depression. We also conducted two focus group discussions comprising 13 of their caregivers. Through a full day stakeholders workshop comprising 15 participants, we drew on the results of our qualitative explorations to identify the pathway to impact of an intervention package (emhGAP-Age) appropriate for the specific needs of persons with late-life depression in Nigeria.

**Results:**

A Theory of Change (ToC) map was produced. It highlights the expected long-term outcomes of emhGAP-Age to include the potential for improvement of the mental health and wellbeing of older people living in Nigeria and the generation of interest among governmental agencies concerned with policy and planning for mental healthcare. Key resources that serve as preconditions were identified to consist of the availability of PHCW who are skilled in the identification and treatment of depression and have interest in and commitment to providing care to older people. Required community resources include support from immediate family, neighbours, and informal groups. Interventions that are appropriate for depression in old age need to incorporate these community resources and address not only the symptoms of the condition but also comorbid physical health problems.

**Conclusion:**

A participatory ToC process led to the identification of the key components of an age-appropriate version of the emhGAP-IG for delivering care to older persons with depression by PHCW in Nigeria.

## Introduction

Late-life depression is associated with complex comorbidities, chronic clinical course, enduring disabilities, high caregiver burden, and stigma(1–3). These factors make the management of late-life depression more complex than depression in other age groups. There is thus a need for an age-appropriate care for late-life depression. This need is particularly urgent in sub-Saharan Africa (SSA) where, partly due to resource scarcity and the stigma attached to mental health consultation, there is a large treatment gap for late-life depression (4, 5).

To bridge the treatment gap for priority mental health disorders in resource-constrained settings, the WHO has developed a generic tool, the Mental Health Gap Action Programme Intervention Guide (mhGAP-IG) for use by non-specialists health care workers (PHCW) to deliver evidence-based treatments for these conditions.(6, 7). Following a consultation exercise to assess the 5-year impact of the mhGAP-IG, the WHO developed and launched the e-version of the mhGAP-IG (v2.0) in October 2017, as an application suitable for both iOS and Android smartphones and tablets. Despite evidence of its feasibility and adoption as a meaningful tool to scale up mental health care(8, 9), the mhGAP-IG has not been evaluated in the specific context of old age.

In the present study, we involved older people in a context appropriate adaptation of treatment approaches in the generic emhGAP-IG which was recently shown by us to be feasible for the care of younger adult depression in Nigerian Primary Healthcare (PHC) facilities (10, 11). Specifically, we: i) conducted formative qualitative research to obtain data from older Nigerians with lived experience of depression as well as their caregivers and, ii) *c*onducted a participatory theory of change (ToC) workshop to frame the results of our qualitative explorations and modelled an intervention package (emhGAP-Age) appropriate for the specific needs of persons with late-life depression in Nigeria.

## Materials and Methods

### Study design

We chose a qualitative design to allow for a more in-depth exploration of the personal experiences and views about late-life depression beyond what might be possible to elicit in a quantitative survey(12). These, in the present study included an understanding of how older people with lived experience and caregivers in the Nigerian context conceptualize and manage late-life depression (e.g., local idioms/understanding of depression), as well as the common care needs of older persons with depression (health, social and functional) which providers need to take account of in planning and delivering care. This information was gathered using Key Informant Interviews (KII) and Focus Group Discussions (FGD).

#### Participants and Settings

The study took place in seven primary health clinics (PHCs) representing a range of urban and rural settings within five local government areas (L.G.As) in Ibadan metropolis, Southwestern Nigeria. In all, Ibadan metropolis has 11 L.G.As and a population of approximately 3.5 million people. There are 186 PHCs in Ibadan each serving a population of approximately 10,000. PHCs in Ibadan and Nigeria are staffed by non-physician primary health workers (PHCW).

#### Procedure

**Figure 1:**
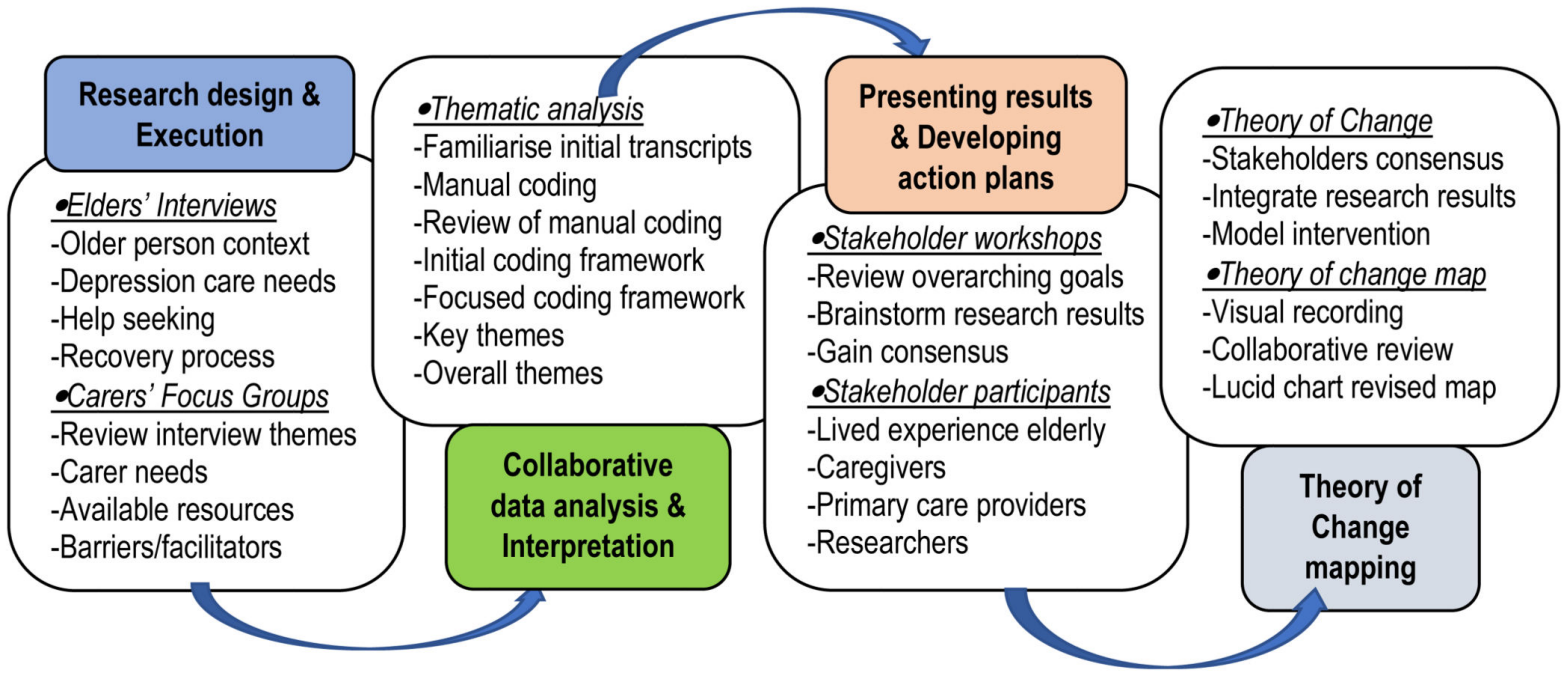
Detail of the study procedures.

Detail of the study procedures are presented in Figure 1.

Participants were purposively selected older (≥60 years) women and men with recent lived experience of depression, as well as their adult (≥ 18 years) male or female caregivers (family or paid). In the context of the present study, paid carers are non-family members who are employed as primary caregiver. All participants provided informed and written consent to participate.

We conducted 15 semi-structured KIIs with older people who had a recent lived experience of depression. This was to explore personal experiences, including sensitive issues that respondents might have difficulty discussing in a group. We also conducted two FGDs with caregivers, comprising thirteen participants in total. The FGDs sought to explore contextual issues of caregiving. Each group included informants purposively selected from across a range of characteristics of caregivers.

#### Topic guides

We developed two contextually relevant interview topic guides for both the FGDs and KIIs (Supplementary Tables I and II). Interview guides were iteratively reviewed by a group of five persons comprising 3 research assistants (RAs) experienced in older peoples’ mental health and services research in Ibadan and Southwestern Nigeria, as well as three experts in older peoples’ mental health, wellbeing and education. The review of KII topic guide took account of the observations of the RAs during of the first two interviews of lived experience older persons. The interviews explored a range of topics such as daily life routines, personal experiences, perception about depression, impact on physical health, help seeking, care needs, recovery process, caregivers’ burden, as well as thoughts about primary care, institutional care and home-based care models for older people with depression. The FGDs and KIIs were conducted between February and June 2022, in Yoruba and English languages, at the PHCs and by trained RA under the supervision of SD and AO. While the KIIs lasted approximately 45 minutes, the FGDs lasted for an hour and half. All sessions were audio-recorded with the permission of participants.

#### Qualitative data analyses

Recordings of FGDs and KIIs were transcribed verbatim by research staff and anonymised to maintain confidentiality. Translation into English was caried out by three independent forward translators fluent in English and Yoruba languages. They compared their versions to identify discrepancies, use of ambiguous or vague wording and accuracy. A final version of the transcript in English was developed with the three original translators by a research staff fluent in English and Yoruba languages. Back translation to the source language was conducted by a fourth independent translator who was blinded to the original transcript.

All final transcripts were analysed in three stages using a reflexive inductive thematic analysis as described by Brown and Clarke(13). Analyses started with three researchers (AO, SD and YF) independently reading and re-reading transcripts for familiarity and to begin to identify pertinent themes. Each researcher labelled meaningful segment of text manually using descriptive codes. The first four KII transcripts were independently coded. The researchers met after each transcript was coded to review their respective preliminary codes to identify areas of similarities and differences. Disagreements in assignment of attribute codes was resolved through discussion to achieve consensus. A draft initial coding framework was then developed, reviewed, and agreed upon.

In the second phase, a further 10 transcripts were coded using the initial coding framework. Systematic collation and review of data within each code was enabled by the computer software package, NVivo 10 (QSR International, 2012). A focussed coding framework was developed in the third phase. Relationships between codes were also identified at this stage. The final phase of the analysis included final interviews, from which four key themes were identified for both lived experience participants and caregivers. These were reduced to three overall themes after review by the research team.

### Theory of change (ToC)

Following the Medical Research Council (MRC) framework(14), we used a participatory ToC approach to frame the results of our exploratory qualitative research and to model an intervention package (emhGAP-Age). Theory of change is an exploration of the theory underlying an intervention to drive change(14). It provides an indication of whether the intervention works, as well as how and why it works within the resources and constraints of the setting of implementation. ToC seeks to equitably involve key stakeholders and researchers in partnership for this purpose through the sharing of expertise. The process allows programme evaluators and researchers to learn about which component of an intervention are most important to drive the expected change. Theory of change in the present study relied on the input of stakeholders from various background during a ToC workshop. We integrated the results of the ToC workshop with results from our qualitative exploratory research.

#### Stakeholders workshop procedure

We organised a full day workshop with stakeholders on 16^th^ November 2022 following standard methodology(15). Stakeholders for the workshop were purposively sampled based on their experience from multiple roles. Fifteen stakeholders participated in the ToC workshop (7 females and 6 males). Their ages ranged from 27-81 years and had a minimum of six years of formal education. Four stakeholders participated in the workshop in their capacity as primary health care workers (1 Nurse, 2 Community Health Officer and 1 Community Health Extension Worker). Two each participated as older people with lived experience of depression (male and female), caregivers (family and paid), representative of lay community (Clergy and Neighbourhood landlord), and researchers (OG and AO). Participants at the workshop also included one PHC coordinator and one social worker. The workshop was facilitated by LK, a medical sociologist who is trained in the facilitation and development of ToC.

The workshop was structured to include an overview of the project, ToC approach, as well as the importance of developing a context appropriate home-based intervention package for the specific needs of persons with late-life depression in Nigeria. The results of our qualitative exploration were summarised and presented to participants. These results were used to elicit structured group discussions and small group exercises. These activities were aimed at creating a ToC map through gaining consensus among stakeholder participants. Points that were perceived as important by the majority of stakeholders were included in the ToC map.

#### ToC map

At the end of the Toc workshop, the facilitator took photographs of the resulting ToC map for the purposes of maintaining a visual record. The first author (AO) subsequently met twice with the ToC facilitator to review the component of the resulting ToC map for internal consistency. A final revised ToC map summarizing the results from the stakeholder’s workshop was constructed by LK using Lucidchart (https://www.lucidchart.com).

## Results

The characteristics of lived experience older people who were interviewed as well as caregivers in the two FGDs are presented in Table 1.

### Key themes from interviews with older people who had a recent experience of depression

Three main themes pertaining to the personal experience of depression in older people were identified by the analysis including: understanding of the experience, causal factors, and enablers of recovery.

#### Theme one: Understanding about depression

This theme is related to older peoples’ personal experience of depression and includes their perception about symptoms, perceived relationship with physical health, and general thoughts about course and prognosis.

##### Perception about symptoms: ‘Sadness of the heart’

Participants described a period lasting several weeks to months when they felt unhappy from within themselves. Many described an overwhelming sadness that would not go away. They described an experience of having no peace of mind, a feeling of despair, being trapped and having no way out. Participants also reported feeling tired constantly during the period of their experience. They described an intense form of weakness that prevented them from carrying out their usual daily activities, including, for some, taking their medications. Participants also reported having sleep difficulties during this period. They described finding it hard to sleep at night or only being able to sleep for a short period at a time, as well as feeling unrefreshed in the morning. Some participants reported they subsequently became tired of life because of these experiences.

‘…, with all of these experiences it will appear as if what is the point of all of my effort, and one will be thinking that this world does not worth it, because you will be thinking that is this the way things will continue, this thought will definitely weaken your system, one can be down after too much thinking and sadness of heart.’

**(Man, 65 years old**)

##### Relationship with physical health

Many participants thought of their experience as indicative of a physical health problem such as malaria, which is thought to be endemic in their environment, but for which they expected to quickly recover from. This perception led to help seeking to treat the suspected physical health conditions. Treatment involved self-medication, or visits to the nearest PHC for those who were able to do so. Many participants reported that they perceived no meaningful improvement in their health status despite treatment for the suspected physical health conditions. Those who presented to PHC and were, through routine physical health checks, identified to have a chronic physical health condition, commonly hypertension and diabetes, perceived that the experience of intense sadness caused them to develop these conditions.

‘I went to the hospital as a result (of the experience of overwhelming sadness) where I was told that I had high blood pressure. So, I think it leads to high blood pressure.’

**(Woman, 60 years old)**

##### Course and outcome: ‘A scar that never heals’

Participants perceived recovery from intense sadness as a sign of restoration of their overall health. However, many valued the absence of symptoms such as sadness, sleep difficulties and lack of energy. They report that improvement in their sleep patterns as well as being able to wake up feeling refreshed in the morning were particularly indicative of their recovery. However, for some participants, there was a perception that recovery from the experience of intense sadness could never be complete. They reported feeling left with a scar that would remain with them forever.

‘It (talking about the depression experience) was a sadness of the heart of the elderly that never dissipates, a scar that never heals.’

(**Woman, 65 years old)**

#### Theme two: Causal factors

This theme is related to the view of the older people with depression about factors associate with depression, including perceived triggers and ongoing concerns.

##### Triggers

Many participants perceived that their depression was triggered by concerns about the wellbeing of their immediate family including own children. Some reported that they were worried about a perceived lack of social and economic progress of their own adult children. These included concerns about lack of academic progress, unemployment for those who were out of school and, for some, a perceived inability for their female children to find a husband. Many expressed feeling embarrassed by these situations. They reported being concerned about the ability of these children to support themselves and their own family in the future. Some perceived being pressured by the weight of responsibility to continue to provide economic support for their adult children despite their old age. Many participants catastrophized about the consequences of not being able to provide needed support their family. For some, they fear that their children may resort to crime if the older person was unable to provide them with economic support.

‘Uhmmm, you see, what caused it was, my very first child, a female, she is supposed to be married but not. My daughter, who is supposed to be married but she is not yet married, it bothers my husband’s mind and mine too.’

**(Woman, 64 years old)**

Participants identified interpersonal conflict with close family members including spouses and adult children as key triggers of intense sadness. Some drew a direct link between marital disharmony and this experience. Other participants reported that they had constant interpersonal conflict with their adult children who, because of economic hardship, were forced to live with and subsist on the goodwill of the older person. These participants linked such conflict to the pressure created by the need to continue to support their adult children in old age. One participant vividly described how some conflict with her children bordered on verbal and emotional abuse.

‘…my son and his wife. They were at Akwa Ibom (Another region of Nigeria) before. They moved in and remained with us for about two years. My husband called them one day and told them that he had done his best and that since they were working, they should begin to fend for themselves. So, my sons' wife thought that I was the one who was behind my husband's decision, which wasn't true. As a result, they (son and daughter in-law) began to hate me. They almost killed me but for the grace of God. I almost died. There was no peace nor harmony. They do take from me, they do collect from me, yet they did not appreciate me. I used to cry a lot after being verbally abused and insulted by my son.’

(**Woman, 71 years old**)

Participants described how a phenomenon of ‘thinking too much’ about problems was the key element in the final common pathway between perceived triggers and ther actual experience of overwhelming sadness. In their views, the perceived triggers led to ‘thinking too much’ about problems which, in turn, led to a possible ‘brain malfunction’ and the subsequent experience of overwhelming sadness.

‘It is not a very good experience, and it is an experience which causes sickness in the body. When someone over thinks about issues it makes the brain to be too occupied making it to malfunction.’

**(Woman, 60 years old)**

##### Ongoing concerns

Some participants perceived their experience of intense sadness was both triggered and made worse by personal economic challenges. They reported that partly because of being unemployed, they were unable to provide basic sustenance, such as food and medications, for themselves and their family during the cause of illness.

For many others, their experience of intense sadness also further worsened their personal economic situations. Many reported being unable to continue their petty trading or subsistence farming work as it became too strenuous to do so. As such they had to resort to selling their household items and other property for survival.

‘Because the thing was troubling me, I had to resign from my appointment. I thought I had to go and rest because of my age. By then, I was already 74. The thing was becoming too strenuous for me, so I wasn't going to work again.’

**(Man, 74 years old)**

Many participants reported that they felt alone during their experience of intense sadness. They reported needing support with activities of daily life such as doing household chores. Many also reported needing support for basic sustenance such as food, as well as payment to access healthcare. Some participants perceived little support from immediate family members or friends, including when they noticed changes in status of the older person. For some, lack of support was because no one was immediately available to do so. Some others reported they still did not receive help form friends and neighbours even when they directly requested. Participants perceived that failure to receive the support they needed during their experience of intense sadness worsened their health condition. Many participants expressed frustration at the prohibitive cost of accessing healthcare and a perceived lack of external support from government to allow the older person access PHC. Those who were able to access PHC reported that basic medical supplies such as drugs needed by the older person were not available at the clinics. They thus expressed a wish for healthcare access and cost of medications to be subsidized.

‘What stressed me was not having any way out, there was no help at all. Everyone I went to told me they can’t help. When they promised to do something, it’s just empty promises, and many of them did not render any help.’

(**Man, 62 years old**).

#### Theme three: Perceived enablers of recovery

This theme is related to older peoples’ views about factors that facilitated their recovery from the experience of depression and includes social enablers such as immediate family, neighbours and faith, as well as healthcare factors such as PHC staff attitude, basic counselling and role of medications.

##### Social enablers of recovery

Participants reported that they were supported to feel better by some family members including their adult children, spouses, and siblings. They reported receiving general words of encouragement such as being told to be strong and to eat well to prevent deterioration of their experience into a longstanding physical illness. Some reported they were advised by their spouses on self-medications with sedatives to improve their sleep, and by friends on herbal concoctions to improve their general wellbeing. Others reported that some siblings who noticed changes in the older persons’ health status supported them to access nearby PHCs either by signposting or providing material support to attend. These participants reported that their spouses supported them to follow the recommended treatment plans from PHC including making sure of dietary and prescribed medications intakes. Some adult children were reported to have assisted the older person with financial support to pay for PHC consultation. Some children were also reported have made frequent telephone and in-person calls to check on the wellbeing of the older person.

‘For my children and in-law, they help. The come to my place to check on us, and when they come, including my son in law who is sitting here with his wife, if there’s no water at home, they will get containers and fetch water for us. They will fill up all the containers in the house with water.’

(**Man, 64 years old**)

Some participants reported that they were supported to feel better by neighbours who noticed a change in their general appearance. They reported being encouraged by such neighbours to visit the PHC. Informal neighbourhood support groups including landlord association were reported by some participants to have made financial donations to enable access to the PHC and pay for consultation and medications. Some individual members of the neighbourhood also volunteered to physically assist patient to get to the PHC.

‘There was also this woman who sold party items in front of my house. When she saw me, she asked what was wrong with me I told her also that I wasn't feeling fine. She then asked me why I didn't go to the hospital; I then told her that I didn't have any money to go to the hospital she then told me to send my account number. It was the lady that sent me 3,000 naira to go to the health centre at Ojoo.’

**(Woman, 60 years old)**

Many participants reported resorting to ‘leaving everything in the hand of God’ when they felt helpless and could not find a way out of their situation. In their view, this decision along with constant prayers and attendance at religious activities helped them to feel better. Some reported receiving counselling and advice from members of the clergy which, in their views, supported them to feel better. Some members of the clergy were also reported to have advised participants to visit PHC. However, many participants attributed their recovery to the answers they received from God for their prayers.

‘Then the church activities too did not allow me to feel it (the sadness) too much, I am a member of Baptist church, there is an association that I belong, the Sunday school section, we see to the preparation of the manual, so I already have the day for the meetings, which I do go to keep myself busy. Also, on Sunday I do attend classes to monitor how the teachers are doing this also help me a lot.’

**(Man, 75 years old)**

##### Healthcare enablers of recovery

Participants reported receiving good quality care from PHC which, in their view helped them to recover faster. They reported being received at PHC with enthusiasm, as well as being attended to promptly. Participants perceived empathy and respect during consultation with the primary health care workers (PHCW). They reported that PHCW allowed adequate time for detailed discussion about the older persons health as well as other concerns. Participants described activities such as counselling, physical health checks and phone call reminders of their appointment as highly valued component of their PHC experience. Participants described counselling sessions as discussive, and with emphasis placed on the need to stop ‘thinking too much’ about problems, importance of prayers and of ‘leaving everything to God’. Participants perceived the discussive nature of the counselling sessions as highly valued. In their views, the sessions provided the older person with an opportunity to socially engage and to share their concerns. Participants also reported that during these sessions, PHCW provided general health education and advice about the role of diet, exercise, and lifestyle factors in the recovery process. Some participants perceived basic counselling and advice as a more effective treatment modality than prescribed medications.

‘For the elderly, comforting discussion is important. Like me, I have no husband or uncle to talk to. Speaking with the elderly is very important. Some people are lonely in their thoughts without having someone to share them with and this can kill. But having a mutual conversation with the elderly, sharing their concerns, is very important.’

**(Woman, 64 years old)**

Participants also reported receiving prescriptions for drugs and injections from the PHC. In the expressed views of participants, the common medications prescribed in PHC when they had presented with overwhelming sadness included antimalarials, medications for blood pressure, as well as mild analgesics for pain symptoms. Some participants also reported receiving PHC prescriptions of ‘drugs for the mind’. Many perceived the prescribed medications as helping them to feel better. However, they report that the cost of medications was prohibitive. Some described experiencing troublesome side effects such as feeling drowsy, dizzy and weak from the use of some medications including antidepressants and sedatives.

‘It was when I came to the health centre that they gave me a drug for the mind. I have been well since then.’

**(Woman, 81 years old)**

### Key themes from caregivers’ focus groups

The analyses of the FGDs generated three main themes around caregivers’ personal experience of caring for an older person with depression and include their views about depression in older people, barriers to caregiving, and implication for a home-based care model (Table 2). There were broad similarities between the views of caregivers and those of lived experienced older people about depression symptoms and perceived triggers (Table 3).

### Outcome of ToC workshop

**Figure 2:**
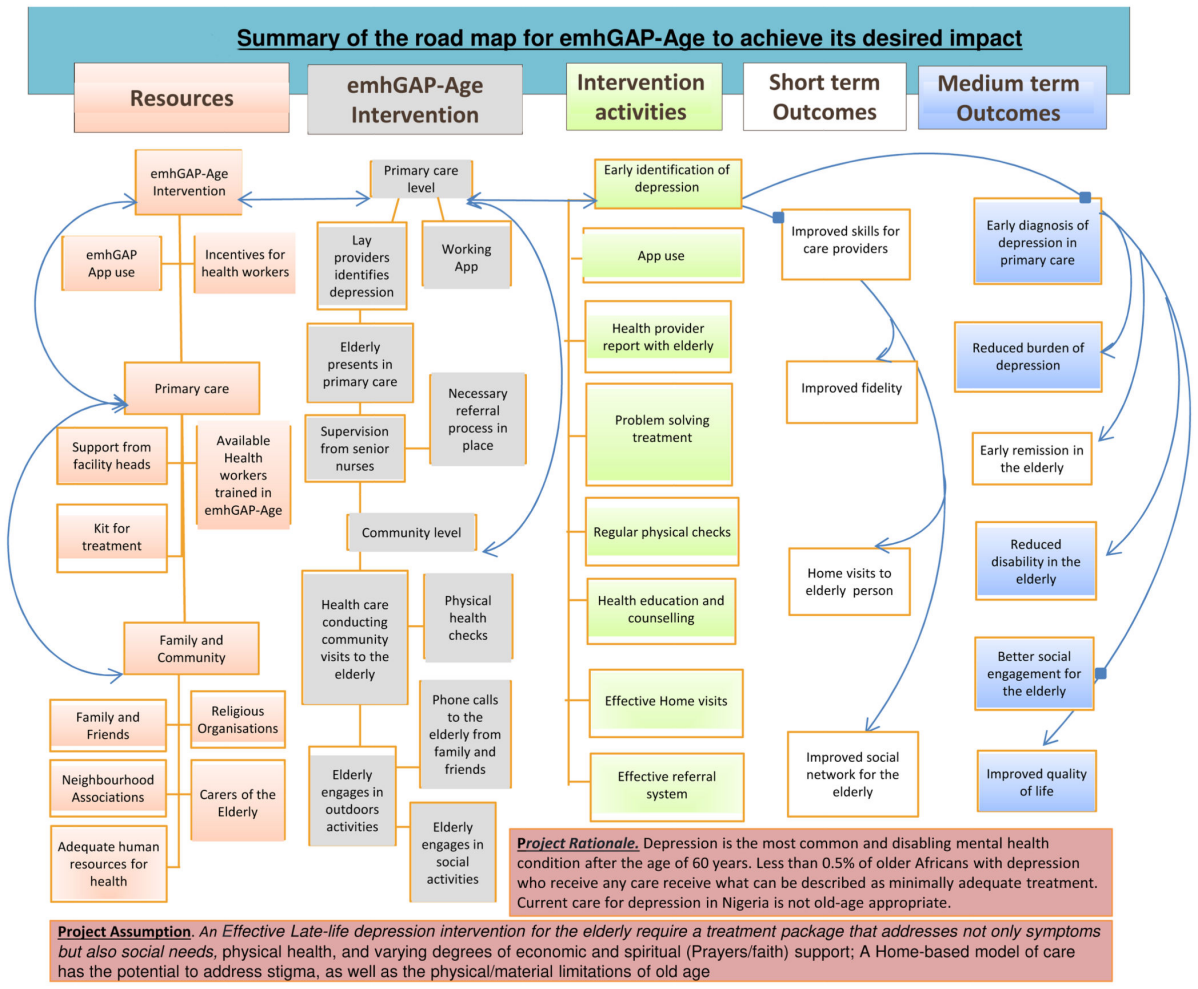
Summary of the road map for emhGAP-Age to achieve its desired impact

Following conventional recommendations for reporting ToC (16, 17), we present in figure 2 the ToC map resulting from the stakeholders’ workshop. The figure summarises the road map for emhGAP-Age to achieve its desired impact.

The consensus of opinions from the stakeholders was that a home-based model of care has the potential to address stigma attached to mental health consultation, and the physical/material limitations of old age. In the view of ToC participants, emhGAP-Age will in the short to medium term bring evidence-based depression treatment to the vulnerable older Nigerian. This was expected to result in early recovery, less disability and improved quality of life. Participants also identified other short to medium term benefits to catchment PHCs and staff, as well as surrounding communities. These, for example include improved clinical skills in the identification and treatment of late-life depression at PHC as well as in the community.

Key resources to drive change were identified in both PHC and community. This according to participants included availability of staff who are skilled in identification and treatment of depression, as well as their generally good disposition to care for older people. Resources in the community include support from immediate family, neighbours, and informal community groups such as landlord association, religious organisations, and members of the clergy. Some of the processes of change identified by participants included better access to evidence-based intervention for depression, better engagement with treatment, support from family and community support organizations, improved social interaction and engagement with others, reduction in social isolation, identification and treatment of comorbid physical conditions through regular health checks, reduction of chronicity and disability, as well as reduction of caregivers’ burden.

The main desired impact of emhGAP-age identified during ToC workshop is the potential for improvement of the mental health and wellbeing of older people living in Nigeria, with possible relevance for other low- and middle-income contexts. The expectation of participants was that a context relevant intervention for late-life depression in Nigeria would generate interest from family groups, advocacy organizations, governmental agencies concerned with policy and planning for mental healthcare, as well as the WHO.

#### Final structure of the emhGAP-Age intervention

The focus of the emhGAP Age intervention is on meeting the most pressing health and social care needs of older people living with depression in Nigeria. Table 4 summarizes the final emhGAP-Age intervention structure.

Basic counselling skills are deployed by community health extension workers (CHEWs) to deliver intervention to older people with depression in their homes where they are also supported by their family, caregivers, and other community organisations. The intervention is delivered in six weekly sessions, as well as monthly follow-up for a total of six months. In the first session, psychoeducation will be used by CHEWs to provide information about the nature of depression in older people, the common symptoms of depression, possible causes, treatability, and the process of treatment.

In the next five sessions, CHEWs use individualised Problem-Solving Therapy (PST-PC)(18) to guide the older person through a stepped process of: 1. Breaking down current problems, difficulties and stressors; 2. Working with the CHEWs to explore and try out options for resolving problems, 3. Use of personal resources (competency enhancement) to overcome problems, 4. Use of available social support systems to overcome problems, 5. Use of wider community resources such as volunteer groups in churches and mosques. In addition to the forgoing, the final session consists of a recap of the process so far: 1. Attempt to consolidate the gains of treatment; 2. Attempt to draw concrete lessons 3. Use lessons to prepare for the future.

The transition between sessions is conditional on achievement of goals rather than specific time points. Patients also receive monthly visits to reinforce improvement on successful completion of the six therapy sessions. Furthermore, remote nurse-led supervision and support of the activities of intervention CHEWs is provided via mobile phone application. This consists of; 1. Documentation of interaction between CHEWs and patients, 2. Documentation of emerging challenges during intervention and how they were resolved, 3. Proper use of referral channels for patients who worsen or become suicidal. First-line Sertraline antidepressants is prescribed by supervisory psychiatrist. This is offered to; 1. Participants with severe depression, and 2. Participants who fail to achieve depression remission at end of the 6-weekly therapy sessions.

## Discussion

Depression is the most common and disabling mental health condition after the age of 60 years (19, 20), and only about 0.5% of older Africans with depression who receive any care receive what can be described as minimally adequate treatment (4). As it is for most mental health conditions occurring in older people, the medical nature of late-life depression is still poorly understood by the population in Nigeria(21). As such, the current care for depression in the country setting is not old-age appropriate (22–24).

The mhGAP-IG or its electronic version has not previously been adapted to the specific context of old age. This is despite evidence of its feasibility and adoption as a meaningful tool to scale up mental health care in primary healthcare settings in Nigeria and other low- and middle-income countries(8–10). We sought in the present study to actively capture the voices of older people with lived experience of depression as well as their caregivers. In the view of ToC workshop participants, an effective late-life depression intervention needs to address not only symptoms of depression, but also able to harness care that involves immediate family and neighbours, economic and spiritual (Prayers/faith) support. Such intervention also needs to pay due attention to older peoples’ physical health needs.

As we previously described (21), the healthcare delivery system in Nigeria is often led by doctors and senior nurses whose jobs are prescriptive and focused on giving instructions to lower cadre staff. There is little opportunity for multidisciplinary contribution in these settings, and the voices of patients and caregivers are often not considered in processes that are meant to benefit them. It is thus not typical in the Nigerian context to involve people with lived experience of mental health conditions or their caregivers in the development of interventions or services(21).

Though desired by patients and caregivers, we found that there were no community-based outreach programmes to ensure continuity of care for older people in the context of the present study. We also did not find existing models of community-based mental healthcare for older people in Sub-Saharan Africa to which our findings could be meaningfully compared. Globally too, the majority of existing community related models of care for older people are focused on those with physical health conditions or their comorbidities, as well as on delaying institutional placements rather than recovery(25). Distinct from these previous interventions, the emhGAP-Age includes the mobilization of existing but previously untapped community resources. In the view of ToC workshop participants, this feature will reduce cost and improve social network as well as social participation of the older person. A home-based model of care also has the potential to address stigma attached to mental health consultation as well as the physical and material limitations of old age.

The current theory driven development of the emhGAP-Age is potentially the first step in bridging existing gap in the continuity of care from primary health care services to community-based care for older Nigerians with depression and other mental health conditions(26). In a previous study of older peoples’ mental health services in Southwestern Nigeria, we found that only 18.4% of patients attended scheduled follow-up care over an approximately 2-year period(26). Over half had dropped out of follow-up after 1-3 irregular contacts with services(26). Factors beyond the control of the older persons such as distance to the primary healthcare centres, mobility issues and reliance on availability of caregivers often led to disengagement from continued care. In the circumstances of high rates of dropout from primary care services and absence of community-based outreach programmes, an integrated model of community-based mental health care that includes health, social, and informal care for older people is needed to ensure continuity of mental health care.

Our reliance on trained CHEWs to deliver emhGAP-Age represents a major advantage for feasibility. This is because the category of health workers are more readily available and often live in the same community as patients(27). Though located in primary healthcare settings, CHEWs are expected to spend 50-70% of their time working in the community. The main limitation of the present study is our reliance on a single ToC workshop in the development of the emhGAP-Age intervention.

### Conclusion

The current participatory ToC process led to the identification of the key components of an age-appropriate version of the emhGAP-IG for delivering care to older persons with depression by PHCW in Nigeria. The suggestion is that a context relevant intervention for depression in older people should give due consideration to the peculiar health and social circumstances of older people. Such intervention must leverage on community resources/groups to support informal care for older people. The emhGAP-Age bridges existing gaps in continuity of care between primary health care services in Nigeria and community-based care for older people with depression and other mental health condition.

## Supplementary Material

Supplementary Tables 1 and 2

## Figures and Tables

**Table 1:** Profile of interview and focus group participants

CHARACTERISTICS	NUMBER	PERCENT
***Older person (N=15)***		
Female	9	60
Married	7	47
Separated but living with family.	7	47
Comorbid physical health condition	9	60
Age (Average and Range)	68	60-81
Education (Average and Range), years	8	0-16
***Caregivers (N=13)***		
Female	9	69
Family carer	9	69
Age (Average and Range)	43	27-55
Education (Average and Range), years	11	0-17

**Table 2:** Summary of themes from exploration of the personal experience of caregivers of older Nigerians with depression

**Views of older people’s depression**	○ **Perception of symptoms** ○ Poor sleep, mood, guilt, memory ○ **Challenging behaviour** ○ e.g., suspicious and uncooperative ○ Seen as a problem interfering with caregiving ○ **Understanding as an inevitable part of ageing** ○ **Identified triggers** ○ Social isolation, ○ Grief, ○ Worry about others, and about own health and economic problems.	*‘What I have noticed is that with respect to the person I'm caring for is that she is forgetful, especially of their phones. And she become unnecessarily worked up as a result.’* **(39 years old daughter carer of an older person with depression)**
		*‘What I notice is that he feels he’s being a bother to us, I really don’t know how to explain it. Since he is incapacitated, he doesn’t want to put burden on us, whoever is helping him. Maybe he wants to take a stroll and I want to follow him; he will say I should not bother. So, he doesn’t like bothering anyone.’* **(55 years old paid carer of an older person with depression)**
		*‘The person I am taking care of is my father. What I think contributes to his depression is if there is no one with or around him. For example, when I and my younger sibling and pastors went to his place on Monday, he was very glad. But as soon as we were ready to take our leave, his mood changed. In essence, when he is alone, he is becoming depressed. But when he sees everybody, he is happy again.’* **(34 years old son carer of an older person with depression)**
		*‘When it first started, she wasn’t sleeping very well. That was how it started. Yes, she was having sleep loss. Then number two, sometimes she will just look at me and say myself and my older sibling connived together to kill her, I will just be laughing at her that nobody is interested in killing you mama. I think old age causes it. As one grows older, a lot of things happen to the body, people like us, we don’t know what we will do when we become aged’* **(55 years old daughter carer of an older person with depression)**
		*‘Sometimes depression is caused by not being able to do as one did when in one's youth and prime of life like engaging in recreational activities or because many of the friends of such depressed elderly ones have passed away. Thinking about their loss and the fact they too will soon pass can cause depression. Also, if the person used to drive but is no longer able to, it can cause depression.’* **(42 years old paid carer of an older person with depression)**
**Barriers to caregiving role**	***Conflicting demands of work and family life*** ***Personal economic challenges*** ***External factors including electricity and transportation*.**	*‘I am a civil servant. I leave home very early in the morning. One must leave home before seven a.m. Initially when she first came to my place (talking about the older person), we employed somebody (paid carer) to take care of her. The person will stay with her when we are not around and do everything, but over the time, the person started stealing. Eventually she left. So, the challenge I have is time, (as well as) getting somebody who is trustworthy who will take care of her, because I have a little time myself.’* **(27 years old nephew carer of an older person with depression)**
		*‘Just as she said, time constraint for us, we also have to build our (own) future, so time constraint is an issue. Sometimes I would have gone out, he might not be able to eat promptly in the afternoon. Before I go out in the morning, I would have prepared breakfast, but lunch is usually late, because I might not be able to return to meet up with two o clock.’* **(55 years old daughter carer of an older person with depression)**
		*‘Just as was mentioned, the financial aspect is another area that limits what I would like to do. There is considerable distance between where I live and where he lives, and I don't have the time to be commuting such distance. If the financial power was there and I was not around, it would just be a matter of asking my younger sibling to go and attend to the needs of my father.’* **(34 years old son carer of an older person with depression)**
		*‘I want to buttress the point just made about money. Money will save time and cover for carer. If I'm well boxed up (have adequate finances), I'd like to have two carers so that they can run shifts instead of me doing that when the one carer I'm paying finishes for the day.’* **(45 years old son carer of an older person with depression)**
		*‘The problem I have at my side is that there is no steady electricity supply, when she gets something to watch throughout the day, I’m sure she will come alive. Sometimes when she is watching a movie when there is power supply, and somebody comes in, she will start telling the person about the movie. It makes her cheerful, but recently there’s been no power supply for about two months now, even my phone there’s no power to charge it.’* **(27 years old nephew carer of an older person with depression)**
		*‘Sometimes I wish I have my own car so that I can drive her to the hospital whenever I feel she needs medical attention. For example, last month, we were coming to the hospital from the house on a motorcycle. When we got to the roundabout, her purse containing her phone fell to the ground and was run over by a vehicle. I was very uncomfortable with that situation. If I have my own car, that situation would have been easily avoidable. I would only need to drive her down to the hospital, see whom we are supposed to see, and drive her back home.’* **(39 years old daughter in-law of an older person with depression)**
**Implications for a homebased care model**	***Dedicated care*** ○ ***Positive views about home-based care*** ○ ***Key enablers of homebased care***	*‘Elderly homes? Though our culture doesn’t accept it as our aged prefer being around by their children. So that’s why it is not easy in Africa.’* **(55 years old paid carer of an older person with depression)**
		*‘About taking them to elderly home to live there, I don’t support it. What we can do is if we want to help our elderly ones, we should employ caregivers for them and for the elderly ones to live where we can be seeing them regularly. That would be the best.’* **(27 years old nephew carer of an older person with depression)**
		*‘Sometimes, when we need to come here and there’s no vehicle to covey us down here, it becomes difficult. I could get fed up and miss the appointment. The stress of boarding or hiring a vehicle is a problem. Concerning your coming to us at home, ah!, we will accept you whole heartedly.’* **(37 years old paid carer of an older person with depression)**
		*‘Such arrangement will help these elderly ones to rest well. If she has a PHC appointment tomorrow, she would not sleep throughout the night until morning, packing her bags and getting ready. By the time she'd be done and waiting for a vehicle to transport her to the hospital, she would be tired. And the trouble of packing is also experienced by the caregivers. But if there is home based medical care, it will be very nice. Maybe they'll be coming three times in a month, and that'll limit her going to the PHC to maybe once a month.’* **(42 years old paid carer of an older person with depression)**
		*‘What I think you can do when you visit us at home is, blood pressure, and so on. You should come with a device to take blood pressure. The drugs he uses. If it is possible to come with such drugs. I think those are the things you can come along with.’* **(37 years old paid carer of an older person with depression)**
		*‘The church gets him involved in its activities like Sunday school. He returns home happy and joyful. If he is unable to go to church, he is visited by the members of the church and they gist and discuss for a while.’* **(45 years old daughter in-law of an older person with depression)**

**Table 3:** Similarities and differences between the views of caregivers and those of lived experienced older people about depression symptoms and perceived triggers.

Themes	Consensus views	Lived experience older adult	Caregivers
**Symptoms**	Physical health^a^ Sleep	Sadness Tiredness Tired of life	Sadness Challenging behaviour Memory Guilt
**Perceived triggers**	Economic problems^b^ Poor social support Worry about others^c^	Interpersonal conflicts	Concerns about own health and dying Loss of autonomy/independence
**Understanding about the experience**	-	‘Sadness of the heart’ ‘Thinking too much’	Inevitable part of ageing

**Note:**

a **Blood pressure and/or body pains**,

b **Also as an ongoing concern**,

c **Mainly own children**

**Table 4:** Basic structure of emhGAP-Age Application

Main modules	Module main purpose	Main data element
**Depression module**	Will give the health worker an overview of depression, common clinical & cultural context of its presentation, signs & symptoms, diagnostic elements, severity criteria, decision nodes	i) Previously contextualized elements of the paper mhGAP-IG & Symptoms counts ii) Severity criteria, Serious suicidal behavior & other Specific needs of older patient iii) Past medical history, current medication, weight, Blood pressure, etc. vi) Social & functional capacity & need for social support
**Treatment algorithm**	Will allow health worker to play different clinical scenarios, treatment options & referral	Adapted algorithm in the generic mhGAP-IG fortified with the culturally-adapted PST-PC, Old age-specific patient treatment needs, links to social support resources
**Support & supervision**	Will enable collection & joint review of patient record & management decisions between CHEWs & supervisors as well as provide for the assessment & enhancement of the clinical competence of the CHEWs.	(i) DASHBOARD-Patients aggregate data and decision summaries (ii) Simple, structured guides, & key monitoring & supervision end-points iii) Content of interaction between CHEWS, older patients & caregivers iv) Challenges during intervention & how these were resolved, v) Specific difficulties requiring additional intervention, Use of referral channels vi) Retention or decay in the acquired skills & competence

## Keypoints

- There is a large treatment gap for late-life depression in sub-Saharan Africa.
- We used participatory methodology to design an age-appropriate depression intervention in Nigeria.
- We identified key resources that may serve as preconditions for depression intervention to drive change.
- A late life depression intervention has the potential to attract policy interest.

## References

[1] Haigh EAP, Bogucki OE, Sigmon ST, Blazer DG (2017). Depression Among Older Adults: A 20-Year Update on Five Common Myths and Misconceptions. Am J Geriatr Psychiatry.

[2] Gitlin LN, Szanton SL, Huang J, Roth DL (2014). Factors mediating the effects of a depression intervention on functional disability in older African Americans. J Am Geriatr Soc.

[3] Fauth EB, Gerstorf D, Ram N, Malmberg B (2012). Changes in depressive symptoms in the context of disablement processes: role of demographic characteristics, cognitive function, health, and social support. J Gerontol B Psychol Sci Soc Sci.

[4] Thornicroft G, Chatterji S, Evans-Lacko S, Gruber M, Sampson N, Aguilar-Gaxiola S (2017). Undertreatment of people with major depressive disorder in 21 countries. Br J Psychiatry.

[5] Ojagbemi A, Gureje O, Bughra D, Moussaoui D, Craig TJ (2021). Oxford Textbook of Social Psychiatry.

[6] World Health Organization (2010). World Health Organization: mhGAP Intervention Guide for mental, neurological and substance use disorders in non-specialized health settings.

[7] Keynejad RC, Dua T, Barbui C, Thornicroft G (2018). WHO Mental Health Gap Action Programme (mhGAP) Intervention Guide: a systematic review of evidence from low and middle-income countries. Evid Based Ment Health.

[8] Oladeji BD, Kola L, Abiona T, Montgomery AA, Araya R, Gureje O (2015). A pilot randomized controlled trial of a stepped care intervention package for depression in primary care in Nigeria. BMC Psychiatry.

[9] Gureje O, Oladeji BD, Araya R, Montgomery AA, Kola L, Kirmayer L (2015). Expanding care for perinatal women with depression (EXPONATE): study protocol for a randomized controlled trial of an intervention package for perinatal depression in primary care. BMC Psychiatry.

[10] Ojagbemi A, Daley S, Kola L, Taylor Salisbury T, Feeney Y, Makhmud A (2022). Perception of providers on use of the WHO mental health Gap Action Programme-Intervention Guide (mhGAP-IG) electronic version and smartphone-based clinical guidance in Nigerian primary care settings. BMC Prim Care.

[11] Taylor Salisbury T, Kohrt BA, Bakolis I, Jordans MJ, Hull L, Luitel NP (2021). Adaptation of the World Health Organization Electronic Mental Health Gap Action Programme Intervention Guide App for Mobile Devices in Nepal and Nigeria: Protocol for a Feasibility Cluster Randomized Controlled Trial. JMIR Res Protoc.

[12] Ojagbemi A (2017). Qualitative and Quantitative Methods of Suicide Research in Old Age. Ann Ib Postgrad Med.

[13] Braun V, Clarke V (2022). Conceptual and design thinking for thematic analysis. Qualitative Psychology.

[14] De Silva MJ, Breuer E, Lee L, Asher L, Chowdhary N, Lund C (2014). Theory of Change: a theory-driven approach to enhance the Medical Research Council’s framework for complex interventions. Trials.

[15] Breuer E, De Silva MJ, Fekadu A, Luitel NP, Murhar V, Nakku J (2014). Using workshops to develop theories of change in five low and middle income countries: lessons from the programme for improving mental health care (PRIME). Int J Ment Health Syst.

[16] Breuer E, Lee L, De Silva M, Lund C (2016). Using theory of change to design and evaluate public health interventions: a systematic review. Implement Sci.

[17] Asher L, Fekadu A, Hanlon C, Mideksa G, Eaton J, Patel V (2015). Development of a Community-Based Rehabilitation Intervention for People with Schizophrenia in Ethiopia. PLoS One.

[18] Chibanda D, Mesu P, Kajawu L, Cowan F, Araya R, Abas MA (2011). Problem-solving therapy for depression and common mental disorders in Zimbabwe: piloting a task-shifting primary mental health care intervention in a population with a high prevalence of people living with HIV. BMC Public Health.

[19] Whiteford HA, Degenhardt L, Rehm J, Baxter AJ, Ferrari AJ, Erskine HE (2013). Global burden of disease attributable to mental and substance use disorders: findings from the Global Burden of Disease Study 2010. Lancet.

[20] Global Burden of Disease Study 2013 Collaborators (2015). Global, regional, and national incidence, prevalence, and years lived with disability for 301 acute and chronic diseases and injuries in 188 countries, 1990-2013: a systematic analysis for the Global Burden of Disease Study 2013. Lancet.

[21] Ojagbemi A, Daley S (2015). Implementing the Dementia Carers Support Initiative of the National Institute for Health and Care Excellence in a sub-Saharan African Setting. J Health Care Poor Underserved.

[22] Leigh-Hunt N, Bagguley D, Bash K, Turner V, Turnbull S, Valtorta N (2017). An overview of systematic reviews on the public health consequences of social isolation and loneliness. Public Health.

[23] The Academy of Medical Sciences (2018). Challenges and Priorities of Global Mental Health in the Sustainable Development Goals (SDG) Era.

[24] Ojagbemi A, Bello T, Gureje O (2021). The roles of depression and social relationships in the onset and course of loneliness amongst Nigerian elders. Int J Geriatr Psychiatry.

[25] Morishita L, Kunz EL, Malone ML, Capezuti EA, Palmer RM (2015). Geriatrics Models of Care: ‘Bringing Best Practice to an Ageing Ameriaca’.

[26] Elugbadebo O, Ojagbemi A, Adefolarin A, Gureje O (2021). Access and Discontinuity of Care at an Outpatient Mental Health Service for Older People in South Western Nigeria. Community Ment Health J.

[27] Ojagbemi A, Gureje O (2020). The importance of faith-based mental healthcare in African urbanized sites. Curr Opin Psychiatry.

