# Supplementary material for: Participatory development of a home-based depression care model with lived experience older Nigerians and their caregivers: a theory of change
Source: Int J Geriatr Psychiatry. Author manuscript; Available in PMC 2023 Nov 10. (PMC7615294; doi:10.1002/gps.6019)
Supplement: Supplementary Tables 1 and 2 [file EMS190589-supplement-Supplementary_Tables_1_and_2.docx]

**Supplementary Table 1: INDIGO: draft interview guidelines for older people with lived experience of depression**

| **S. N** | **THEMES AND POSSIBLE PROMPTS** | **FURTHER PROBES** |
| --- | --- | --- |
| X | **General –**  **C**an you tell me a little bit about yourself/and how you spend your time |  |
| 1. | **Understanding about depression** |  |
| 1.1. | *Interview:*  You have had some contact with the primary health clinic –  How has your health been in the past one month? | (If not answered by response, explore –  – can you tell me about your understanding of the experience ‘’feeling *low in your mood and feeling tired of everything around you?*  What were your thoughts about these experiences?  How has it affected your health?  What do you think could be the causes of these experiences?  -and why?  Are there any kinds of stressors that made your experience worse?  -and why? |
| 2. | **Help seeking** |  |
| 2.1. | *Interview:*  Can you tell me more about what led to you seeking some help during your experience of being *‘low in mood and feeling tired of everything around you’?* | If not answered by response, explore –  How did people at home, friends or others respond when they noticed changes in you?  -how were they of help?  What led to visiting the primary health clinic? |
| 3. | **Impact on physical health** |  |
| 3.1. | *Interview:*  How is your physical health? | In what ways did your experience of being *‘low in mood or feeling tired of everything’* affect your physical health?  What kind of support are you currently receiving in regard of maintaining physical health?  [Probe more about specific experiences? |
| 4. | **Recovery** |  |
| 4.1 | *Interview:*  What does recovery from your experience of being *‘low in mood or feeling tired of everything’* mean to you?  What supported you to feel better? | (If not answered by response, explore –  How are you feeling now?  What has changed?  What support did you receive from the clinic?  What support did you receive from others?  In a perfect world, if anything else could be done what would this be? |
| 5. | **Perception about home-based care** |  |
| 5.1. | *Interview:*  We are planning to develop a homebased treatment model that would allow a primary health care worker to provide the treatment and support needed for older people who are experiencing low mood, low energy or having *“little interest, or pleasure in doing things”* (or equivalent term).  What are your thoughts about this idea? | (If not answered by response, explore –  What would be helpful to include in a home-based treatment for an older person who is having the experience of being ‘*low in mood or feeling tired of everything*?’  [views on features and why they are likely to be helpful?]  [explore more about examples]  (If not answered by response, explore –  Think about any other areas of your life beside medical care, what else would be important to include in your ideal home-based care? |
| 6. | **Perception about smartphone-based support** |  |
| 6.1. | *Interview:*  What are your views about the use of smartphones? | What do you know smartphones can be used for?  [Explore more about specific experiences and examples]  What are your thoughts about the idea of a healthcare worker providing you with support and healthcare with the aid of mobile application?  [ Explore more about participant perception, reaction, and concerns] |

**Thank you for your time**

**Supplementary Table 2: INDIGO: Draft Focus group guidelines for caregivers of older people with depression**

**Aim of focus group:**

*1. To understand the support needs of family carers of depressed older people*

*2. To understand how family carers define depression in older people*

*3. To understand how support needs of family carers might be met*

*4. To understand family carer views about the INDIGO model*

| **S.N** | **THEMES AND POSSIBLE PROMPT** | **FURTHER PROBES** |
| --- | --- | --- |
| 1. | **General** |  |
| 1.1 | *Focus group:*  We invited you to this meeting because you have been supporting your family member whilst they have been unwell (Depression or ‘*low in mood* *and tired of everything)*  Can you tell me how you help him/her? | What has the experience of supporting your family member been like?  [Probe more about specific experiences]  What difficulties have you experienced in helping him or her?  [Probe more about specific examples] |
| 2. | **Understanding about depression** |  |
| 2.1. | *Focus group:*  Can you tell me about your own understanding of the experiences of the family member you are supporting? (e.g., feeling ‘*low in mood* *and tired of everything)* | What are your thoughts about these experiences?  What do you think could be the causes of these experiences?  -and why?  Are there any kinds of stressors that make this kind of experiences worse?  -and why? |
| 3. | **Barriers to care of an older person with depression** |  |
| 3.1. | *Focus group:*  Thinking about your role in supporting your family member who is having these experiences (Depression or ‘*low in mood* *and tired of everything*)……  Is there anything which affects your being able to support him/her? | Probe in areas of:   - Are there other factors (*may or may not be related to him or her)* that you would see as hindering your being able to help? |
| 4. | **Facilitators of care of an older person with depression** |  |
| 4.1. | *Focus group:*  What factors would you see as helping your being able to support your family member who is having these experiences (Depression or ‘*low in mood* *and tired of everything*)? | What other factors (*may or may not be related to him or her*) would you see as helping your being able to support him/her? |
| 5. | **Support for caregivers** |  |
| 5.1. | *Focus group:*  Are you receiving any help in your role in supporting your family member who is having these experiences (Depression or ‘*low in mood* *and tired of everything*)? | *Probe in areas of:*  Support received from the clinic.  Support from other family members  Support received from other persons/organisations.  [With specific examples] |
| 5.2. | *Focus group:*  How has the help you currently receive affected the support you can give to your family member who is having these experiences (Depression or ‘*low in mood* *and tired of everything*)? | In a perfect world, if anything else could be done to support you, what would this be?  How has the support you **DO NOT** currently receive hindered your being able to help your family member who is having these experiences (Depression or ‘*low in mood* *and tired of everything*)? |
| 6. | **Perception about home-based care and needs of older people with depression** | |
| 6.1. | *Focus groups:*  We are planning to develop a new way of working where a healthcare worker would come to the home of people who have this problem (Depression or ‘*low in mood* *and tired of everything*) and provide treatment.  What do you think about this? | What are the common needs of people like your family member who is having these experiences (Depression or ‘*low in mood* *and tired of everything*)?  [Explore views on needs and why including them in a home-based care package would be helpful?] |
| 6.2. | *Focus group:*  The healthcare worker might also use a tablet or smart phone,  What you think about this? | Apart from medical care, think about any other areas of your role in supporting your family member who is having these experiences (Depression or ‘*low in mood* *and tired of everything*)?  What else would be important to include in your ideal home-based care? |

**Thank you for your time**
